# Supplementary material for: Interaction between Bacteria and the Immune System for Cancer Immunotherapy: The α-GalCer Alliance
Source: Int J Mol Sci. 2022 May 24;23(11):5896. doi: 10.3390/ijms23115896 (PMC9180740; doi:10.3390/ijms23115896)
Supplement: Supplementary file 1 [file ijms-23-05896-s001.zip › ijms-1737278-supplementary.pdf]

Table S1. Association between Stages and OS for all species.

COAD			
**Condition**	**p-value**	**n present**	**n absent**
Bacteroides fragilis: present vs. absent	0.6	241	195
Bacteroides vulgatus: present vs. absent	0.5	286	150
Prevotella copri: present vs. absent	0.4	123	313
Bacteroides fragilis: present vs. absent in tumor stage I	0.9	41	32
Bacteroides vulgatus: present vs. absent in tumor stage I	0.9	55	18
Prevotella copri: present vs. absent in tumor stage I	0.2	18	55
Bacteroides fragilis: present vs. absent in tumor stage II	0.09	96	70
Bacteroides vulgatus: present vs. absent in tumor stage II	0.9	108	58
Prevotella copri: present vs. absent in tumor stage II	0.4	53	113
Bacteroides fragilis: present vs. absent in tumor stage III	0.8	64	60
Bacteroides vulgatus: present vs. absent in tumor stage III	0.2	78	46
Prevotella copri: present vs. absent in tumor stage III	0.2	36	88
Bacteroides fragilis: present vs. absent in tumor stage IV	0.3	35	30
Bacteroides vulgatus: present vs. absent in tumor stage IV	0.04	41	24
Prevotella copri: present vs. absent in tumor stage IV	0.1	16	49

READ			
**Condition**	**p-value**	**n present**	**n absent**
Bacteroides fragilis: present vs. absent	0.6	60	98
Bacteroides vulgatus: present vs. absent	0.5	85	73
Prevotella copri: present vs. absent	0.5	44	114
Bacteroides fragilis: present vs. absent in tumor stage I	0.2	13	19
Bacteroides vulgatus: present vs. absent in tumor stage I	0.4	24	8
Prevotella copri: present vs. absent in tumor stage I	0.4	6	26
Bacteroides fragilis: present vs. absent in tumor stage II	0.5	14	34
Bacteroides vulgatus: present vs. absent in tumor stage II	0.9	21	27
Prevotella copri: present vs. absent in tumor stage II	0.2	14	34
Bacteroides fragilis: present vs. absent in tumor stage III	0.4	20	27
Bacteroides vulgatus: present vs. absent in tumor stage III	0.9	22	25
Prevotella copri: present vs. absent in tumor stage III	0.2	15	32
Bacteroides fragilis: present vs. absent in tumor stage IV	0.5	10	16
Bacteroides vulgatus: present vs. absent in tumor stage IV	0.3	14	12
Prevotella copri: present vs. absent in tumor stage IV	0.2	8	18
